# Case Report: Subcapsular hematoma post-transarterial embolization and its laparoscopic resolution

**DOI:** 10.3389/fmed.2025.1710345

**Published:** 2026-01-07

**Authors:** Haizhao Yi, Qi Wang, Xuecong Bai, Jinlong Liu, Hua Fu

**Affiliations:** Department of Hepatobiliary Surgery, Affiliated Hospital of Chengde Medical University, Chengde, Hebei, China

**Keywords:** case report, complication, hepatic hemangioma, laparoscopic caudate lobectomy, subcapsular hematoma, transarterial embolization

## Abstract

**Background:**

Hepatic hemangioma (HH) is a common benign liver tumor. Transarterial embolization (TAE) is a widely adopted minimally invasive treatment. Subcapsular hematoma (SCH) is an exceptionally rare and severe complication following TAE, with only sporadic cases reported and no standardized management protocol.

**Case presentation:**

A 43-year-old female presented with acute right upper quadrant pain 8 days after an attempted TAE for a caudate lobe HH at another institution, which had been aborted due to unsuccessful vessel cannulation. Upon admission, contrast-enhanced CT revealed a large SCH in the right hepatic lobe alongside the known caudate lobe HH. Her condition rapidly deteriorated, with a significant decline in hemoglobin and signs of hemorrhagic shock, necessitating emergency intervention. She subsequently underwent a simultaneous laparoscopic procedure consisting of evacuation of the massive SCH (approximately 2000 mL) and enucleation of the giant caudate lobe hemangioma. The surgical strategy included a left-sided approach, utilization of the Arantius ligament as a key anatomical landmark, and low central venous pressure (CVP) anesthesia to minimize intraoperative bleeding.

**Conclusion:**

This case represents, to our knowledge, the first report of SCH as a complication following hepatic artery angiography or attempted TAE and the only reported case of simultaneous laparoscopic management of both a ruptured SCH and a caudate lobe HH. It highlights that SCH, although rare, can be a life-threatening complication of endovascular procedures. Laparoscopic surgery is a feasible and effective therapeutic option for managing such complex conditions. This report underscores the critical importance of meticulous technique during TAE and vigilant postoperative monitoring to prevent this serious complication.

## Introduction

Hepatic hemangioma (HH) is the most common benign solid tumor of the liver, typically congenital or estrogen-dependent, usually asymptomatic, and slow-growing (1). It is generally a well-circumscribed hypervascular lesion with no potential for malignant transformation (2). According to previous studies, one of the main surgical indications for HH is a diameter greater than 5 cm, primarily because hemangiomas larger than 5 cm are more likely to cause abdominal symptoms (3). In recent years, transarterial embolization (TAE) has been widely used as a minimally invasive treatment for HH (4). TAE functions by obstructing the blood supply to the lesion using chemotherapeutic agents. Common complications include pain, nausea, fever, liver abscess, sepsis, and hepatic artery dissection (5).

However, subcapsular hematoma (SCH) has never been reported as a post-TAE complication. Potential mechanisms may include intraoperative puncture-related injury to hepatic arterial branches, inappropriate postoperative anticoagulation, or local ischemia leading to increased hepatic parenchymal fragility. The literature on SCH following TAE is limited to sporadic case reports and lacks systematic analysis. This case provides a detailed account of the diagnosis and treatment of SCH after TAE for HH, suggesting that clinicians should pay close attention to the operative standards during TAE (such as avoiding excessive embolization and controlling the volume of embolic agents) and strengthen postoperative monitoring (including imaging follow-up and assessment of vital signs).

## Case presentation

**Figure 1:**
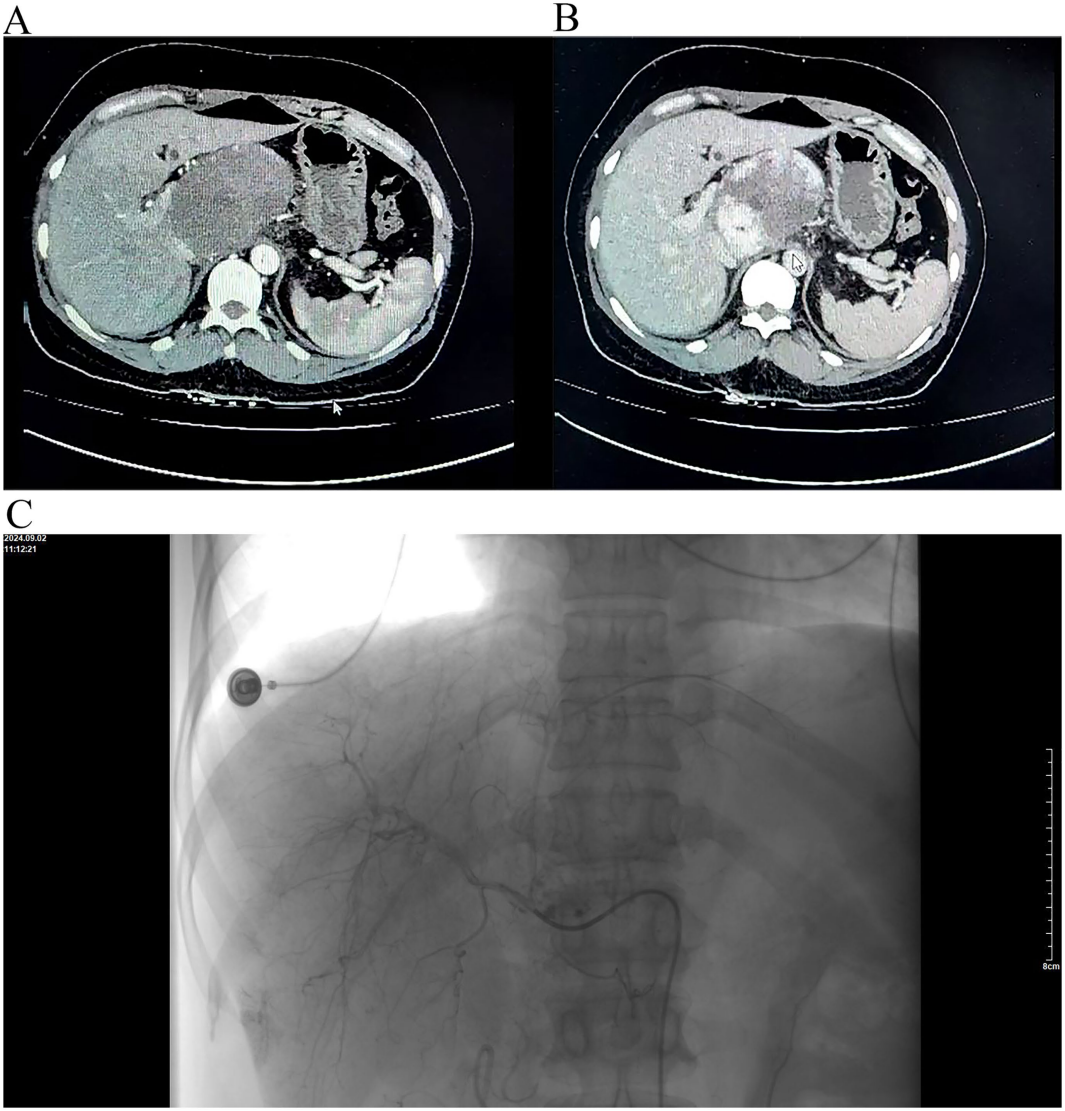
**(A)** Arterial phase of contrast-enhanced CT demonstrates a giant hemangioma in the caudate lobe. No hepatic capsular abnormality is evident. observed. **(B)** Venous phase of contrast-enhanced CT. **(C)** Hepatic arteriography confirms the presence of the hemangioma; however, no definite feeding vessel or subcapsular hematoma is identified.

**Figure 2:**
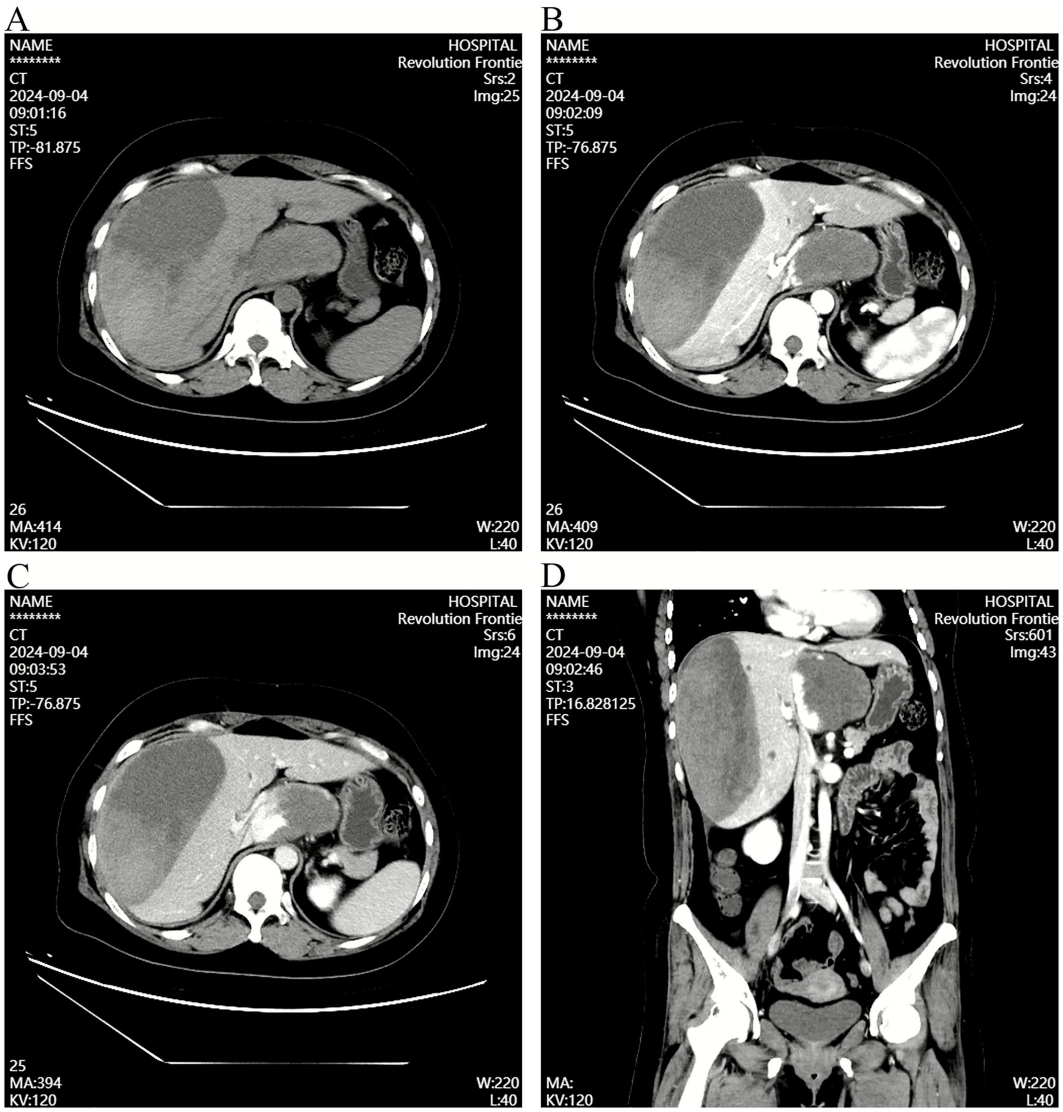
Emergency contrast-enhanced CT revealing subcapsular hematoma and hepatic hemangioma. **(A)** Unenhanced CT phase showing the subcapsular hematoma and caudate lobe hemangioma. **(B)** Arterial phase highlighting the subcapsular hematoma and hemangioma. **(C)** Venous phase depicting the subcapsular hematoma and hemangioma. **(D)** Coronal reconstruction of contrast-enhanced CT clearly displaying the subcapsular hematoma and caudate lobe hemangioma.

A 43-year-old female experienced sudden-onset persistent dull pain in the right upper abdomen 8 days prior, without obvious triggers, accompanied by radiation to the shoulder and back. The pain was aggravated by respiratory movement and changes in posture, with no significant relieving factors. She reported frequent episodes of non-bloody nausea and vomiting, with temporary symptom relief after vomiting. She denied chills, fever, chest tightness, jaundice, or abnormal bowel or bladder habits. One day before admission, abdominal contrast-enhanced CT performed at Weichang Manchu and Mongol County Hospital revealed a hepatic hemangioma. Hepatic artery embolization (TAE) was attempted but failed because hepatic arteriography demonstrated no identifiable artery supplying the hemangioma in the caudate lobe (Figure 1). Seven hours after admission, her abdominal pain intensified. The patient presented to the Emergency Department of the Affiliated Hospital of Chengde Medical College for further evaluation and treatment. Emergency contrast-enhanced CT demonstrated hemorrhage within a lesion in the right hepatic lobe and the HH in the caudate lobe (Figure 2).

Physical Examination on Admission: Vital signs included a temperature of 37 °C, blood pressure of 113/83 mmHg, heart rate of 88 bpm, and oxygen saturation of 98%. Abdominal examination: Epigastric tenderness was noted without rebound tenderness or rigidity. Shifting dullness was negative, and bowel sounds were normal (4/min). Laboratory Findings: Complete blood count: Hemoglobin 89 g/L, RBC 2.79 × 10^12^/L. Liver Function Tests: ALT 179.00 U/L, AST 169.00 U/L. Total bilirubin, amylase, renal function, and coagulation profiles were within normal ranges.

The patient received fluid resuscitation, anticoagulation, immobilization, antibiotics, and cardiac monitoring. At 6 h post-admission, she developed worsening abdominal pain, lethargy, and progressive declines in blood pressure, hemoglobin, and RBC count, indicating a ruptured hepatic hematoma with active intra-abdominal bleeding and impending hemorrhagic shock. An emergency exploratory laparotomy was performed.

**Figure 3:**
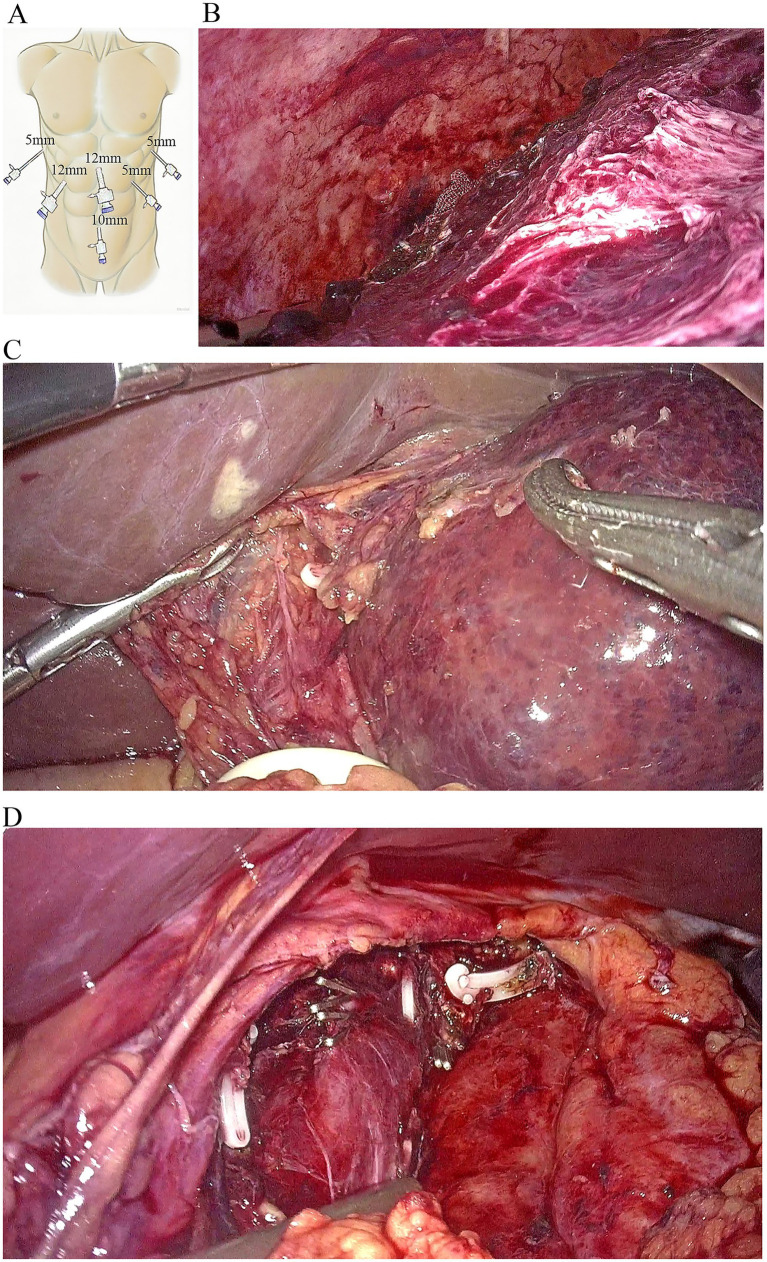
Laparoscopic evacuation of hepatic hematoma and caudate lobectomy. **(A)** Trocar placement configuration for laparoscopic evacuation of the hepatic hematoma and caudate lobectomy. **(B)** Surgical field following complete evacuation of the subcapsular hematoma. **(C)** Exposure of the left margin of the hemangioma after mobilization of the left hemiliver. **(D)** After complete resection of the hemangioma, the inferior vena cava is clearly visualized.

The specific surgical procedure was as follows. A pad was placed under the right shoulder to achieve a 30° tilt, and pneumoperitoneum was established with the patient in the head-up, legs-separated position. Because the patient also had a cavernous hemangioma in the caudate lobe, the port layout was designed to facilitate resection of both the hematoma in the right hepatic lobe and the hemangioma in the caudate lobe. During resection of the right hepatic hematoma, the operating table was tilted 45° to the left. For mobilization of the caudate lobe, it was rotated 45° to the right. Pneumoperitoneum was maintained at 12–14 mmHg, and central venous pressure (CVP) was controlled below 5 cmH₂O. Under laparoscopic guidance, five additional trocars were placed as operating ports (Figure 3A).

This port configuration provided optimal access for the chief surgeon and the first assistant to mobilize the right liver, permitting bilateral dissection of the caudate lobe. Dark-red hepatic parenchyma with a massive subcapsular hematoma measuring 20 cm in diameter was identified, protruding from the right hepatic surface. An 8 cm hemangioma was observed in the caudate lobe beyond the liver surface, posing a significant risk of spontaneous rupture. Preoperative CT imaging did not provide definitive evidence of active bleeding from hepatic artery branches. Therefore, the hematoma capsule was incised using an ultrasonic dissector, and approximately 2000 mL of accumulated blood was aspirated. The entire hematoma capsule was excised. The operative field was irrigated with normal saline and inspected carefully to identify the bleeding source. Meticulous hemostasis was achieved using bipolar electrocautery, and no bile leakage was observed (Figure 3B).

**Figure 4:**
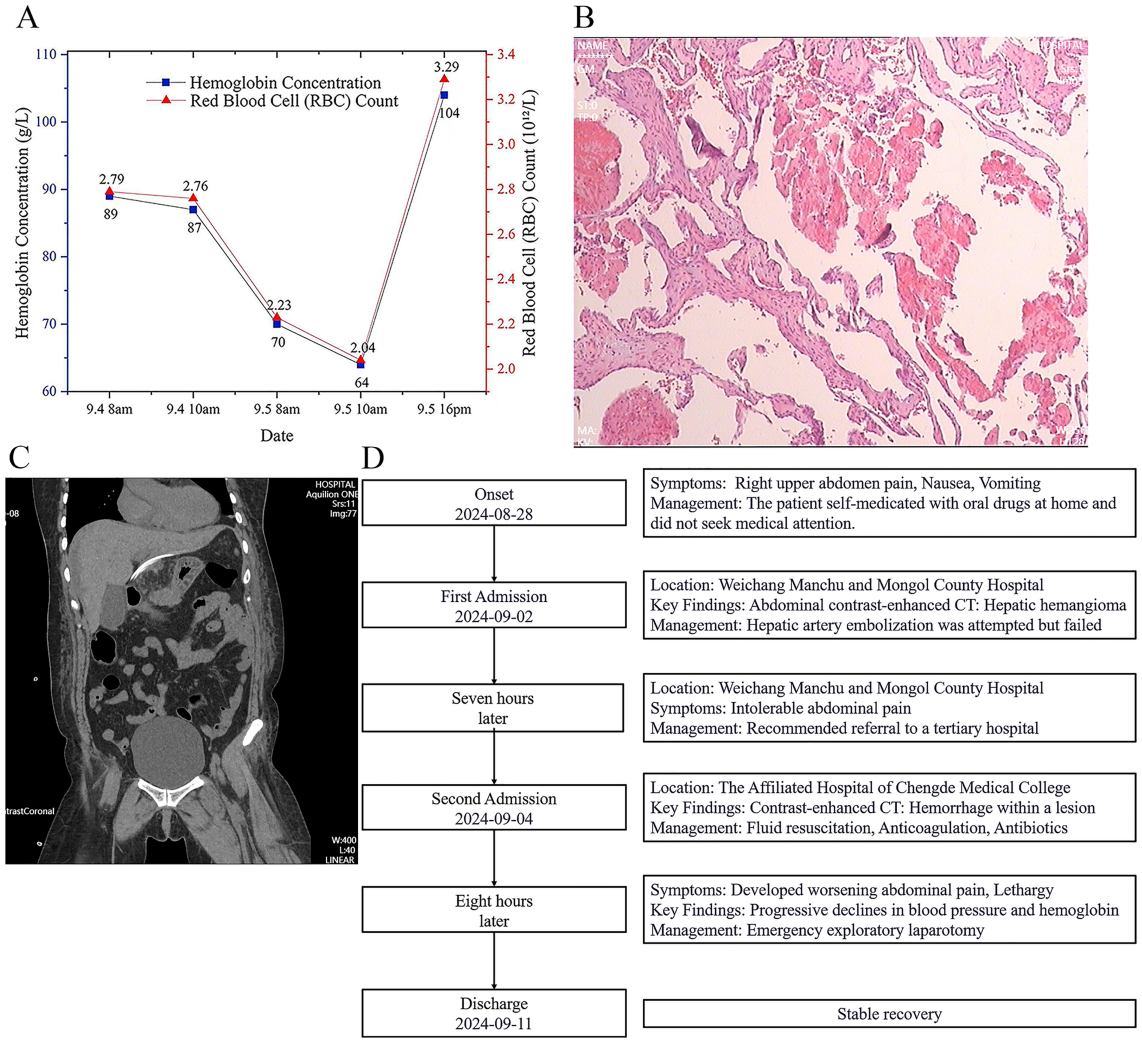
Postoperative recovery and histopathological findings. **(A)** Trends in hemoglobin levels and red blood cell counts during hospitalization. **(B)** Postoperative unenhanced CT scan demonstrating complete removal of the hematoma and hemangioma, with no signs of intra-abdominal bleeding. The abdominal drain is visible *in situ*. **(C)** Histopathological examination confirming the diagnosis of cavernous hemangioma. **(D)** The timeline of the clinical course for the patient.

Following hemostasis and stabilization of vital signs, laparoscopic enucleation of the caudate lobe hemangioma was performed. Initially, the round ligament, falciform ligament, left coronary ligament, and left triangular ligament were divided. The left lateral lobe was fully mobilized and retracted superiorly and to the right to facilitate exposure of the caudate lobe (Figure 3C). The lesser omentum was opened to expose the caudate lobe, and a Pringle maneuver tourniquet was preplaced. The third hepatic portal was dissected, and the short hepatic veins between the hemangioma and the inferior vena cava were sequentially clipped and divided. The hepatic vein and portal vein branches supplying the hemangioma in the caudate lobe were then transected. Subsequently, parenchymal transection of the hemangioma was carried out along its margin from the caudal to the cranial direction until the hemangioma was completely resected (Figure 3D). The patient received antibiotics, hepatoprotective agents, prothrombin complex concentrate, and fluid support. Follow-up blood tests demonstrated improvementin hemoglobin (Figure 4A), and histological examination showed a main vascular tumor with a fibrous capsule (Figure 4B), The postoperative abdominal CT on day 4 revealed no abnormalities (Figure 4C). The patient was discharged on September 11 with stable recovery (Figure 4D). At the 7-month follow-up after discharge, the patient had recovered well, with stable hemoglobin levels and an asymptomatic abdominal examination.

## Discussion and conclusion

To the best of our knowledge, this is the first documented instance of SCH occurring as a complication following hepatic artery angiography. It also represents the only reported case in which both a hepatic hematoma and a caudate lobe hemangioma were simultaneously resected using a laparoscopic approach. The hepatic capsule and liver parenchyma in the right lobe were widely separated due to hematoma formation. It is presumed that the guidewire inadvertently injured vessels, including branches of the hepatic artery supplying the hepatic capsule, during hepatic artery angiography. Such unintentional vascular injury may lead to rupture of the hepatic capsule and parenchyma, resulting in subsequent hemorrhage. Another hypothesis is that the guidewire traversed the hepatic artery, penetrated the hepatic parenchyma, and injured microscopic branches of the portal or hepatic veins within the subcapsular tissue, ultimately leading to a subcapsular hematoma. These findings align with the known pathophysiological mechanism leading to subcapsular liver hemorrhage following ERCP (6). However, hepatic artery angiography did not reveal hematoma or isolated blood vessels supply to the hepatic capsule. Nevertheless, the patient had no history of liver cirrhosis and exhibited low portal and hepatic venous pressures. Under such conditions, venous bleeding typically does not result in massive hematoma formation (7). Additionally, a non-guidewire-related mechanical injury, specifically excessive traction on the arterial catheter, can induce subcapsular hepatic parenchymal injury, leading to the rupture of small vessels and subsequent formation of a subcapsular hematoma (8). Furthermore, the high-pressure injection of contrast medium may cause rupture of intrahepatic microvessels during hepatic arteriography, which can also be a potential cause of subcapsular hepatic hematoma (9). Since this patient underwent emergency surgery, a comprehensive evaluation could not be performed; therefore, the possibility of spontaneous subcapsular hepatic rupture and hemorrhage following hepatic arteriography cannot be excluded. Nontraumatic SCH may occur in severe preeclampsia, hemolytic anemia, elevated liver enzyme levels, low platelet count syndrome, or ruptured hepatic tumors (10). Emerging evidence indicates that targeted SARS-CoV-2 infection may trigger spontaneous subcapsular hepatic hematomas, possibly mediated through virus-induced endothelial injury and coagulopathy (11). Iatrogenic subcapsular hepatic hematoma is exceedingly rare, with incidence markedly lower than that of spontaneous etiologies (12–15).

The clinical manifestations of SCH are diverse, including abdominal pain, anemia, hypotension, and fever (6). Recent literature and statistical analyses identify abdominal pain as the most common symptom, followed by hypotension, anemia, and fever, whereas chest pain and dyspnea are relatively uncommon (16). Laboratory tests may only demonstrate decreased hemoglobin, requiring imaging examinations for diagnostic confirmation. Contrast-enhanced CT provides the highest diagnostic value and can accurately define the size, location, and characteristics of SCH. Treatment planning for SCH depends on clinical symptoms, physical findings, and hemodynamic stability. Common management strategies include conservative treatment, TAE, and surgery (17). A literature review reported that conservative treatment accounted for the highest proportion of SCH cases at 39.3%, followed by surgery (27.9%), percutaneous drainage (22.5%), and TAE (8.2%) (18). For patients with mild symptoms, stable hemodynamics, and no signs of peritoneal irritation, observation is recommended (19). Monitoring should include reassessment of hemoglobin levels and repeat abdominal ultrasound or CT to evaluate changes in clinical status.

In the present case, the clinical condition deteriorated with a sustained decrease in hemoglobin and indications of active bleeding or enlargement of the SCH on imaging. Interventional embolization or surgical treatment was therefore considered. Although interventional approaches are effective for subcapsular hepatic hematoma, they fall short of achieving curative resection of the underlying hepatic hemangioma. Prior to admission, the patient underwent unsuccessful hepatic artery embolization at Weichang Manchu and Mongol County Hospital. In addition, a hematoma may compress the liver parenchyma, causing severe abdominal pain, hemorrhagic shock, secondary infection, or liver failure (20). Without timely intervention, a second operation may be required to remove the hematoma or perform partial hepatectomy, substantially increasing patient burden. Consequently, the decision was made to proceed with timely laparoscopic surgical intervention.

Laparoscopic techniques have advanced to allow resections in all liver segments; however, hepatectomy in challenging locations, particularly laparoscopic resection of the caudate lobe, remains a major difficulty in laparoscopic local liver tumor surgery. In this case, the complexity also lay in the laparoscopic resection of a giant hemangioma located in the caudate lobe. As the deepest and most anatomically complex liver segment, the caudate lobe is bordered anteriorly by the hepatic artery and portal vein of the first hepatic hilum, posteriorly by the large-caliber inferior vena cava, and cranially by the hepatic veins of the second hepatic hilum (21). Moreover, laparoscopic caudate lobectomy (LCL) was introduced relatively late and still lacks standardized procedures. The first successful LCL was reported by Dulucq et al. in 2006 in a patient with a metastatic colon cancer lesion in the caudate lobe (22).

Laparoscopic and open surgery each have their respective advantages and disadvantages in the management of hepatic rupture. Several studies have indicated that laparoscopic resection remains safe and feasible even for large lesions with a high risk of bleeding in hepatic rupture (23–25). However, open surgery remains indispensable in certain scenarios, particularly for cases requiring extensive hepatectomy or complex reconstruction (26). Furthermore, open surgery enables more rapid intervention and management of intraoperative contingencies, such as gas embolism (27).

In the present case, the hemangioma was massive, occupying the entire caudate lobe and causing significant mass effect, which rendered exposure of the third hepatic portal extremely difficult. A left-sided approach was therefore selected. Using the Arantius ligament as the boundary between the left lateral lobe and the Spiegel lobe, the Arantius ligament was divided first to facilitate a wider rightward opening of the transection plane. This maneuver enabled full exposure of the left margin of the caudate lobe hemangioma and allowed gradual enucleation of the lesion. Branches of the hepatic and portal veins supplying the caudate lobe were divided, followed by transection of the short hepatic veins of the third hepatic portal once the resection surface had been adequately exposed.

In conclusion, this case highlights the critical importance of recognizing subcapsular hepatic hematoma as a potential, albeit rare, complication following angiographic procedures such as transarterial embolization. It represents the first documented instance of subcapsular hepatic hematoma formation after hepatic artery angiography and the only reported case employing a simultaneous laparoscopic approach to evacuate a massive hematoma and resect a caudate lobe hemangioma. The successful application of advanced laparoscopic techniques demonstrates their feasibility and efficacy in the emergent management of complex, multifocal liver pathologies. Furthermore, this report emphasizes the need for heightened procedural vigilance during endovascular interventions, including precise guidewire manipulation and controlled administration of embolic agents, together with stringent postoperative monitoring to reduce this serious risk. This study provides important insights into the management of iatrogenic liver injury, underscoring the role of minimally invasive surgery as a definitive therapeutic approach for these complex cases.

### Lessons learned

The rapid advancement of minimally invasive techniques has enabled the treatment of most abdominal diseases through such approaches. For liver tumors, particularly those located in the caudate lobe, laparoscopic surgery offers definite therapeutic efficacy, minimal trauma, and faster recovery, demonstrating significant advantages over other modalities. However, it remains technically demanding with a high skill threshold. The management of caudate lobe hemangioma necessitates rigorous preoperative evaluation and careful selection of treatment strategy, as inappropriate therapeutic choices may substantially increase both the physical and economic burden on patients.

## Data Availability

The datasets presented in this article are not readily available because of ethical and privacy restrictions. Requests to access the datasets should be directed to the corresponding authors.
